# Physical and Biological Determinants of Collective Behavioural Dynamics in Complex Systems: Pulling Chain Formation in the Nest-Weaving Ant *Oecophylla smaragdina*

**DOI:** 10.1371/journal.pone.0095112

**Published:** 2014-04-23

**Authors:** Thomas Bochynek, Simon K. A. Robson

**Affiliations:** Centre for Tropical Biodiversity & Climate Change, School of Marine and Tropical Biology, James Cook University, Townsville, Queensland, Australia; Arizona State University, United States of America

## Abstract

The evolution of nest weaving, the inclusion of larval silk in the nest walls, is considered one of the pinnacles of cooperative behaviour in social insects. Within the four ant genera in which this has evolved, *Oecophylla* are unique in being the only group that precedes the deposition of larval silk by actively manipulating the leaf substrate to form a nest chamber. Here we provide the first descriptions of the manipulation process within a complex-systems framework. Substrate manipulation involves individual ants selecting, grasping and attempting to pull the edge of the substrate. These individuals are then joined by nest mates at the work site, who either select a site beside the first individual or grasp the body of the first or preceding worker to form a chain of pulling ants that together drag and bend the substrate. Site selection by individual workers is not random when confronted with an artificial leaf, with individuals more likely to grasp a substrate at its tip rather than along a more broad edge. The activity of additional individuals is also not random, with their activity being grouped in both space and time. Additional individuals are more likely to join an existing biting individual or pulling group. The positive feedback associated with the early stages of pulling behaviour appears typical for many of the collective actions observed in social insects.

## Introduction

Social insects are prime examples of collective systems in which numerous relatively simple individuals can together display highly diverse and adaptive group-level behaviours [1]–[3]. Numerous studies of social insects exploring these processes include the selection of new nest sites in *Apis* [4] and *Temnothorax* [5], [6], the dynamics of foraging in army and mass recruiting ants [7], [8], nest building in wasps [9] the regulation of nest temperature in *Apis* [10], retrieval group size in *Formica schaufussi* [11] and adaptive search in termites [12]. These studies have not only formed the basis of theoretical advances in our understanding of the organisation and evolution of key attributes of social insect colonies such as division of labour and individual behavioural specialization [11], [13]–[15] but have been applied more broadly to issues understanding and optimizing the decision-making capabilities of groups as a whole, independent of the social insects (e.g. [16]–[19]).

In parallel with the recognition that individual simplicity can underlie group complexity and the role of positive and negative feedback, has come the understanding that the exact form of many of the complex group level phenomena represents the interaction between relatively simple individual rules and the biotic (and abiotic) environment in which they are enacted. The type of mound structure constructed by the black garden ant *Lasius niger*, the shape of the royal chamber around a termite queen and the foraging pattern of army ants most likely reflect changes in the physical structure of the substrate (moisture, airflow and prey distribution) rather than changes in the behaviour of individuals themselves [1], [20], [21]. The importance of nest construction and self-assemblages to many social insect species suggest that studies of such processes, though relatively under explored, play a key role in understanding the dynamics of collective action [22].

Weaver ants *Oecophylla smaragdina* represent an ideal system to examine the organisation of collective behaviours associated with nest construction, in particular the role of physical factors (reviewed in [23]). An individual colony can occupy many trees with highly divergent leaf types, yet they still manage to construct nests [24], presumably with a relatively simple and consistent set of individual behavioural rules. Nests are constructed by pulling leaves together and gluing them in place with larval silk. Workers physically join together to form two types of chains that are key elements in nest construction: ‘hanging’ or ‘bridging’ chains to cross a gap between leaves, and/or ‘pulling’ chains used to bring two substrate surfaces together [22]. Studies of the recruitment dynamics involved in chain formation highlight the roles of negative and positive feedback and support the notion that models of complex systems such as self-organisation are applicable [25], [26].

Despite being considered to represent a pinnacle of cooperative achievement in social insects [27] the behaviours associated with the basic elements of nest construction and the role of the physical substrate remain almost entirely unknown. Sudd [24] described the manipulation of leaves to make nests with a terminology similar to that now used in the study of complex systems, but unfortunately offered no empirical evidence in support. Individuals were described as randomly choosing sites to bite and pull, with differences in leaf flexibility mediated by a greater attraction of workers to a bending site (now termed positive feedback) ultimately determining the final shape of a nest.

In this paper we explore individual and collective behaviours associated with pulling chain formation in *Oecophylla smaragdina*. What are the decision rules used by individuals to initiate pulling chain formation - do they choose the substrate randomly and are individuals attracted via positive feedback to active sites? How does the physical environment influence the dynamics of substrate manipulation via the formation of pulling chains, and what are the implications for our understanding of the organisation of collective decision-making systems?

## Methods

### Establishment of *Oecophylla* groups

Experimental groups of ants were constructed from ten *Oecophylla* nests collected on the campus of the James Cook University in Townsville, Australia (19°19′42S 140°45′30E). Nests were placed in a laboratory fridge at 8°C for one hour to reduce the worker mobility, and a subgroup of approximately 500 workers and brood was selected from each nest to form discrete experimental groups. Groups were kept in white plastic containers 30 cm deep, 50 cm long and 30 cm high, with fluon-coated walls to limit escape. A retort stand (35 cm high) and clamp was placed in the container and later used to hold an artificial nest substrate. Workers and brood that were not allocated to experimental groups were returned to their original collection site.

All groups were kept in a climate controlled room at 27±1°C, with the humidity at 75±5% during daytime (8 am–5 pm), and 22±1°C and 50±5% humidity during night (5pm–8am) for a maximum of one week. During this time they were supplied with water and diluted honey *ad libitum*, and freshly killed crickets every two days. Groups were released at their collection site after a maximum of one week in the laboratory.

### Experimental design

The basic experimental design involved moving an experimental group into an observation arena (a wooden box 120 cm deep ×60 cm wide ×120 cm high, with a light source placed centrally above the setup, the interior surfaces painted white, and a small hole in the end wall allowing access to a video camera lens) followed by the attachment of the artificial substrate to the retort stand. All experiments were initiated between 8 am and 10 am, each experiment was run for a maximum of 8 hours before being terminated, and experimental groups were maintained for at least 24 hours before the start of each experiment. Experiments were conducted from November 2010 to February 2011, the entire duration of experiments were recorded with a tripod mounted Sony HDR-XR150 camera, and videos observed with QuickTime Media Player.

**Figure 1:**
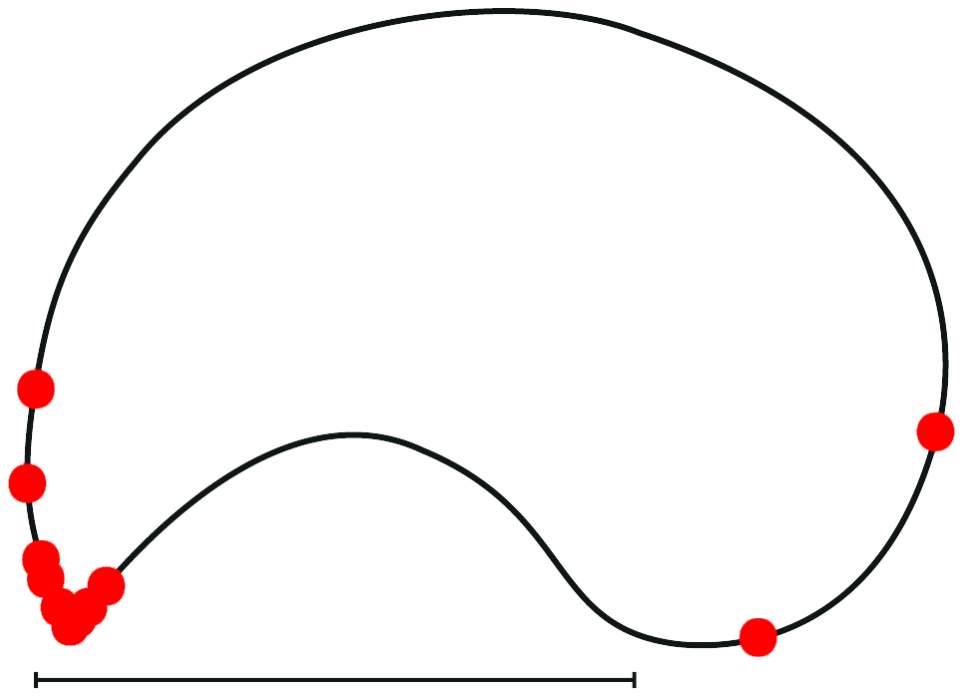
Location of first bites on artificial substrate. Location of the first bites in each of the 13 trials in which general nest construction activity occurred is indicated on the reproduction of an artificial leaf. The majority of bites occurred immediately on the tip itself. The scale bar at the bottom of the image measures 10

In order to investigate the effects of the physical substrate shape on the location of bites and chains, experimental groups were offered a ‘ying-yang’ shaped substrate, which possessed only a single tip (Figure 1) constructed from white A4 bond paper as an artificial leaf. In these cases 24 hours elapsed between trials. The time and position of all bites and chains were noted for each trial, and the orientation of the substrates (tip pointing to the left or right) was randomized to control for any potential direction effects. Each artificial leaf was used in only a single trial.

### Experimental analysis

To determine the effect of the substrate shape on the formation of pulling chains, the substrate was divided into two regions of equal perimeter length: one region containing the tip and the other containing the opposite rounded edge. Also, the observed probability that the location of the first bite in each trial was on the half of the artificial leaf containing the tip was compared to an expected probability of 0.5 using a Binomial test.

To determine if the orientation of the substrate influenced pulling chain formation, the frequencies with which the first bite occurred in either the tip or rounded region of the substrate was compared between those substrates in which the tip was facing either the left or the right, using a Fisher's exact test.

The spatial and temporal dynamic of bites within the context of individual trials was determined using two randomizations, in which the value of a test statistic generated using the experimental data was compared to the values likely to be obtained through 10,000 randomisation trials where individuals were randomly allocated to either spatial or temporal units. The number of individuals in the randomisation trials matched the number of individuals in the respective experimental trial. To determine if the distribution of bites was randomly distributed or clumped in space, the perimeter of the artificial leaf used in each trial was divided into 36 equidistant sections of 1 cm length and the number of bites in each section recorded for each individual trial. The frequency distribution from each trial was then compared to a uniform distribution (calculated by dividing the total number of biting individuals in the trail by the number of spaces, 36) and the test statistic, in this case a chi-square statistic, calculated. The randomisation process involved randomly allocating the same number of individuals in each trial to a spatial position, calculating the same test static as above, and repeating for a total of 10,000 randomisations. The significance of the test lies in the probability of obtaining the observed test statistic under a random model, not in how it was generated per se. To determine if individual bites were clustered or randomly distributed in time, the video recording of the entire trial was divided into intervals of 15 seconds duration and the number of bites occurring in each 15-second interval recorded. Observed and expected chi-square values were calculated as above, with the exception that individuals were randomly assigned to the appropriate number of temporal rather than spatial units.

Two approaches were taken to visually represent the dynamics of chain formation over time. The first involved recording the time and location at which an individual ant bit the substrate, and the time and number of any additional individuals grasping a previous ant to form a chain. An individual biting ant was therefore considered to represent a chain size of 1, and several biting individuals and/or chains were considered to be in a single biting ‘group’ if the bites or chains occurred within 5 mm (approximately twice worker head width) of an existing biting ant or chain. The timing, duration and number of ants in biting chains and groups were then examined with ribbon graphs, in which the position of the ribbons reflects the order of bites and chains on the artificial leaf.

In the second approach we used the software ImageJ to produce an animated video that highlighted the location of ants on the substrate and how this location changed with time and pulling activity. Still images of the activity on the artificial leaves were taken from the original video every 5 seconds. In an automated procedure, ants were identified in every image through a colour threshold mask that effectively discarded all background information. The resulting image was converted to a black and white figure and filtered until the resulting outline provided an estimate of the individual's body size. The density of black pixels of these idealised images was then visualized using the ImageJ 3 d surface plot function, which produces colour images where colour intensity reflects the number of ants on the substrate. The function parameters were chosen in such a way that distinction of both individual ants as well as strong clusters would be possible on the resulting images. An example of the procedure is given in the Results section. These thus generated 5-second snapshots were then combined into an.avi video file with a frame rate of 2 images per second that summarises the activity of workers on the substrate in time lapse.

All statistical analysis was performed using the software TIBCO Spotfire S+8.2.

## Results

Nest construction activity consisted of individual workers biting the perimeter of the nest substrate and, through walking backwards, attempting to pull it inwards. Chain formation typically involves additional individuals grasping the gaster of the ant in front of them that is either already attached to the substrate or another pulling ant. Parallel chains can also form next to existing chains. Once a sufficient number of individuals are involved in chains at a particular site, the perimeter of the substrate is then pulled towards the centre of the artificial leaf, forming a potential nest chamber. We refer to this process as ‘substrate rolling’.

In a total of 51 experiments conducted, general nest construction activity was observed in 13 experiments. Of these, substrate rolling occurred in seven experiments. While substrate rolling and chain forming was therefore not a rare occurrence in a laboratory setting, it occurred on the reverse side of the artificial substrate in four of seven experiments exhibiting substrate rolling, precluding detailed video analysis via the elevated camera.

Individual ants do not select bite locations randomly. The locations of the first bites observed in each of the 13 trials in which biting occurred were not distributed evenly between the tip and the round side of the artificial leaves; instead, eleven of the 13 initial bites were located on the tip side compared to only two on the round side (Binomial test, p = 0.022, Figure 1).

There was no bias in whether the first bite was located on the tip or round side as a function of the orientation of the tip side of the artificial leaf (left or right). The first bites were located on the tip and the round side respectively in four and one of the five cases in which the tip of the substrate was pointed to the left and biting occurred, and in seven and one of the eight trials in which the substrate was pointed to the right and biting occurred (Fisher's exact test, p = 1.0).

Within the context of an individual trial in which biting, chain formation and rolling occurred, there was significant clustering of bites in both space and time. Summaries of the temporal and spatial dynamics of individual bites for the three nests that could be analysed with video analysis (Nest A = 51 bites, Nest B = 39 bites and Nest C = 15 bites) are shown in Tables 1 and 2. In all three nests, the observed chi-square values for the temporal organisation of bites during each trial were greater than the expected chi-square values generated after 10,000 randomizations based on the expectation of randomness over time. In two of the three nests (Nest B and C), the observed chi-square values for the spatial organisation of bites during each trial were always greater than the expected chi-square values generated after 10,000 randomizations based on the expectation of randomness over time. In the remaining Nest A, the observed chi-square value was equal to a single estimate based on 10,000 randomizations, suggesting conservatively that the probability obtaining the observed value under randomness was less than 1 in 1,000. In all cases, the variance-mean ratios of >1 indicates that the data are clumped both temporally and spatially: individual ants are more likely to bite the substrate next to an existing biting ant, and at a similar time to this previous ant.

**Table 1:** The dynamics of nest construction activity (bites per 1 cm perimeter) is temporally clustered.

	Nest A	Nest B	Nest C
Observed chi-squared value	237.87	163.28	645.38
Maximum simulated chi-squared value	233.31	154.77	594.50
Mean	0.44	0.51	0.04
Variance	0.91	1.02	0.06
Variance-to-Mean ratio	2.07	2.01	1.59
p-value	p<0.0001	p<0.0001	p<0.0001

**Table 2:** The dynamics of nest construction activity (bites per 1 cm perimeter) is spatially clustered.

	Nest A	Nest B	Nest C
Observed chi-squared value	79.59	100.38	117.00
Maximum simulated chi-squared value	79.59	78.23	78.60
Mean	1.42	1.08	0.42
Variance	3.22	3.11	1.39
Variance-to-Mean ratio	2.27	2.87	3.34
p-value	p<0.001	p<0.0001	p<0.0001

**Figure 2:**
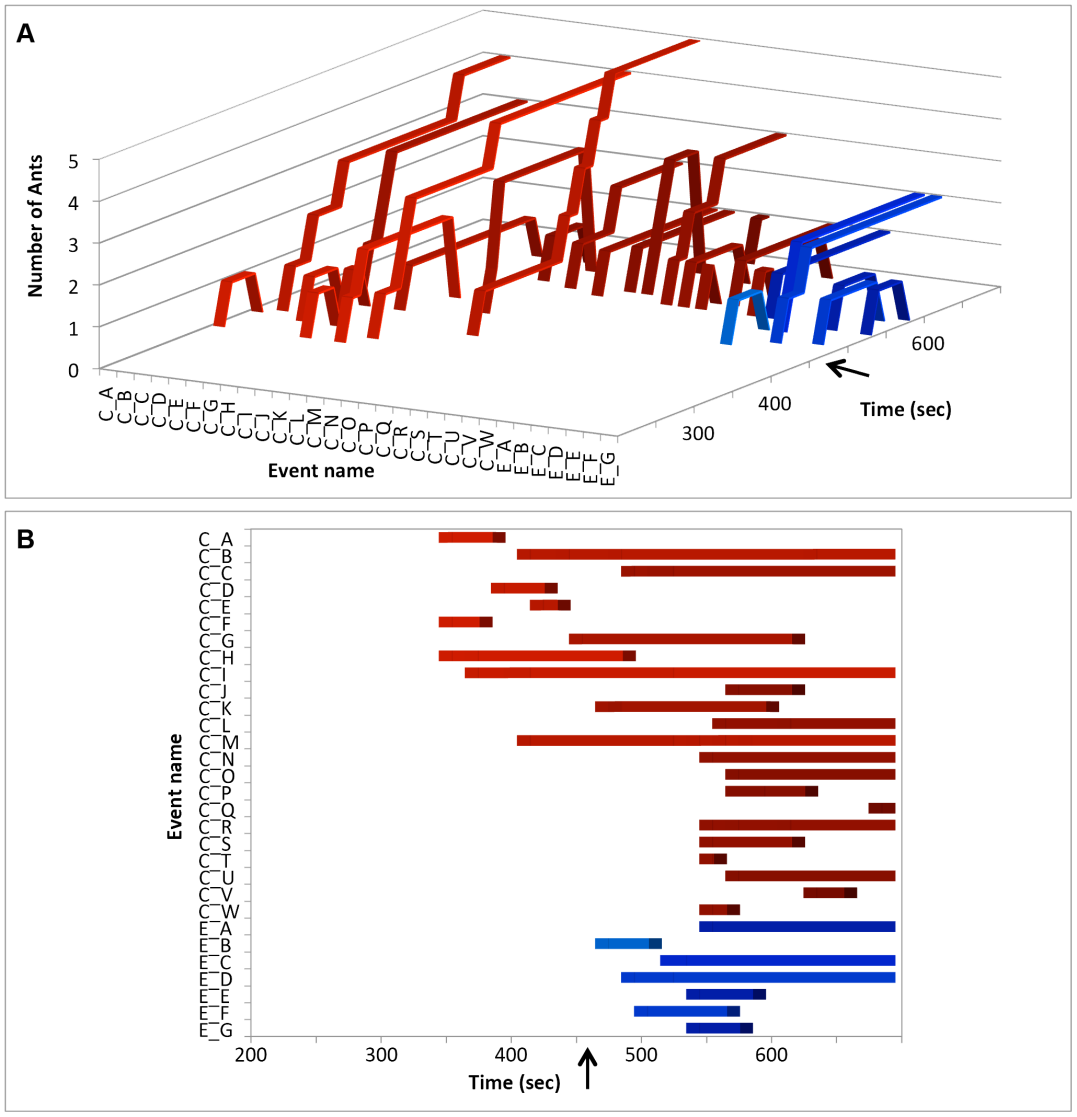
Spatial and temporal dynamics of bite and chain formation in Nest B. Each bite/chain is represented by a unique ribbon. The spatial arrangement of the ribbons reflects their proximity on the edge of the substrate. The bites/chains are organised in two groups, C and E. The black arrow indicates the time at which group C starts rolling up the substrate. Figure 2A highlights the different numbers of workers recruited to each chain, while Figure 2B gives a top-down view of the same graph to exemplify the different time individuals persist.

A visual representation of the dynamics of biting and chain formation is shown for Nest B, in Figure 2, which indicates the timing, location, duration, size and group identity of 31 of the 39 bites/chains that formed during this trial. Of these 39 bites and chains, two groups (group C and group E), with 24 and seven bites/chains respectively were distinguished. In the first group, bites C_A, C_F and C_H formed together approximately 350 seconds after the start of the experiment at the tip of the artificial leaf. The individuals forming bites C_A and C_F left after 30 and 40 seconds respectively, while C_H built up to two ants. They were joined by additional chains within 5 mm proximity, named C_G and C_I. A second group of bites/chains (Group E) formed at the rounded edge of the artificial leaf 470 seconds into the experiment and eventually comprised seven distinct bites/chains. Group C started rolling the tip of the substrate back towards the middle of the artificial leaf 460 seconds after the start of the trial (indicated in Figure 2 by a black arrow). The second group (E) had not managed to roll the substrate by the end of the trial.

**Figure 3:**
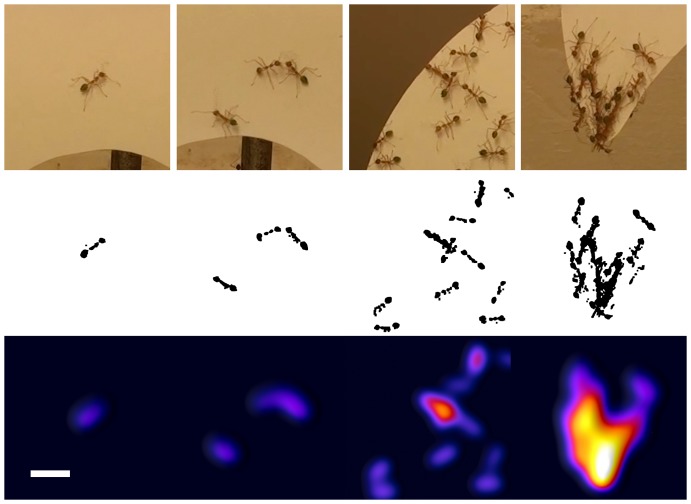
Example of image processing procedure. The first row of images shows extracts from the video recording of the experiments, with different densities of ants. The same images are shown in the second row after having been submitted to a colour threshold filter and subsequently filtered to remove appendages. The final sequence shows colour images depicting the density distribution of ants on the previous pictures. The white scale bar at the bottom left of the figure measures 1“Experimental Analysis”.

**Figure 4:**
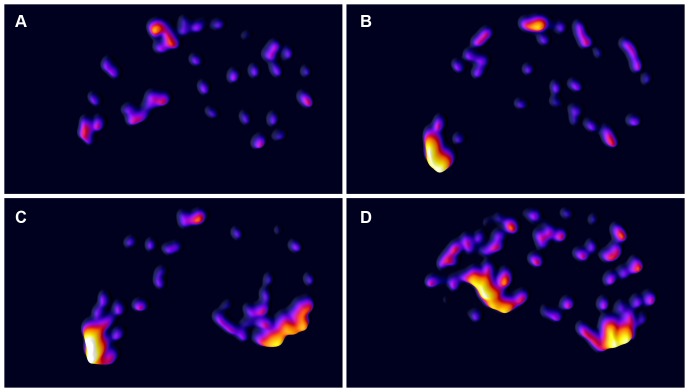
Dynamics of substrate rolling. Display of the density distribution of ants on the artificial leaf at four time stages throughout the experiment using Nest B. The perimeter of the leaf is not indicated but is highlighted by the distribution of ants in each image. For an impression of how the colour intensity relates to the actual number of ants in a given area, please refer to Figure 3. The tip of the ying-yang shaped artificial leaf is to the left. At time A, shortly after the insertion of the artificial leaf, ants are exploring the substrate. At time B, 400 seconds after the experiment commenced, a group of ants has formed at the substrate tip. Chain formation and tip rolling are evident at time C (470 seconds) and D (600 seconds) respectively.

To emphasise the density of workers on the artificial substrate, still images were generated from the video recorded during the experiment as outlined in the section Experimental Analysis. Figure 3 shows a sample of the procedure, depicting extracts from the original images, the processed black and white representation of the ant position, and a colour image illustrating ant density. The figure illustrates the process of generating idealised visualisations of ant densities, and serves as a scale for Figure 4 and Video S1.

Video S1 shows the complete sequence of Nest B, which together with Figure 4 highlights the dynamic nature of the chain formation process. In the early stage of the Nest B trial ants are distributed relatively evenly across the surface of the artificial leaf (Figure 4A). Approximately 400 seconds after the start of the trial a group of ants (group C, Figure 2) has formed at the tip on the left side of the artificial leaf (Figure 4B). After 470 seconds from the start of the trial the second group (group E, Figure 2) forms in addition to the first group (Figure 4C). Figure 4D, approximately 600 seconds into the experiment, shows the progress of group A rolling the tip towards the centre of the artificial leaf, which began at 460 seconds.

## Discussion

The ant genus *Oecophylla* contains the only two ant species known to collectively form chains of living individuals that pull and modify the substrate in order to form a communal nest [23]. Yet despite being considered to represent a unique feat of cooperative achievement in social insects [28] this behaviour remains relatively unexplored. The formation of pulling chains has been previously examined within the general context of the construction of arboreal nests [24], [28] and the evolution of nest-weaving within ants as a whole [27]. The consensus of these studies has been that individuals select work sites randomly, that the physical attributes of the substrate are likely to play a key role in determining where ants actually nest and what final shape the nest takes, and that work sites that are bending may be more attractive to workers than sites that are not. These ideas of group formation and collective choice with their elements of randomness and positive feedback hint at the applicability of more recent studies of complex systems and collective choice in social insects (e.g. [1]), conceptual techniques that have been applied to such aspects of *Oecophylla* behaviour as the formation of hanging chains and their ability to ‘choose’ and bridge gaps [25], [29].

By offering groups of *Oecophylla* workers artificial substrates, we have demonstrated that the selection of the initial sites for pulling chains is not random. The first individual to bite the substrate in each nest is more likely to select the tip of a substrate than a more rounded margin and subsequent individuals are attracted to those already doing so (individuals biting the edge of the substrate are clumped in both space and time, see Tables 1 and 2). Additional workers subsequently join these individuals to form chains. While it is possible that the location of a chain at a tip may also influence the probability that individuals will subsequently join this chain, the detection of chains forming and growing at non-tip sites indicates that the presence of individuals in a chain can in itself be a sufficient stimuli to attract additional workers. The formation and growth of these ‘non-preferred’ sites are shown in Figure 2, bite group E, in blue, and Figure 4, which shows group E forming at the bottom right of the substrate.

The descriptions of pulling chain formation described here are in partial agreement with the earlier descriptions of Sudd [24]. *Oecophylla* workers are attracted to active successful work sites that are already bending, a process that is likely to be amplified through a typical process of negative and positive feedback as individuals abandon non-bending work sites and join areas where leaves are bending. The initial selection of sites by the first worker to attempt substrate manipulation however is not random, rather, these individuals are themselves more likely to select narrow tips rather than broad margins of the substrate.

Pulling groups can themselves be comprised of multiple parallel chains of workers working in concert, though the participation of individuals in these groups, and the duration of individual chains, can be highly dynamic (Figure 2). A single pulling event typically comprises multiple chains that persist for varying durations and it is relatively common for multiple pulling groups to form and effectively compete with each other. The extent to which individual chains might compete with each other within the context of a single pulling event is unknown.

Although individual ants are more likely to commence pulling chain formation on a substrate tip, we do not yet know if individuals are making this choice based on the physical characteristics of the substrate *per se*. Understanding the perceptions and decision rules of individuals represents a key step in understanding this and other dynamic systems, as individuals may be responding to other cues such as the need to slow and turn around at a tip rather than perceiving the actual tip itself. The ant *Formica schaufussi* for example matches the size of the retrieval group to prey mass [30] without individuals perceiving the magnitude of the task required (the size of the prey item and the number of ants required to retrieve it). Rather, individuals respond in a binary manner classifying prey as being individually retrievable or not, with group size matching occurring during the retrieval process itself [31], [32].

The demonstration of positive feedback mechanisms (the attraction and clumping of biting workers in space and time), the dynamic nature of the pulling group (pulling groups do not always form at the tip and multiple pulling groups can form) and the likelihood that negative feedback processes - such as the loss of attraction of workers to sites that are not moving - occur, suggests that the processes increasingly found to underlie collective action in social insects - randomness, positive and negative feedback [1] - are also found to affect pulling formation in weaver ants. These findings also support the idea that environmental heterogeneities, when overlaid with a relatively simple set of individual rules, may play a key role in determining the outcomes of the collective processes we see [21].

## Supporting Information

Video S1 **Dynamics of substrate rolling in Nest B.** Colour intensity represents the density of ants on the artificial substrate. The substrate shape is not explicitly indicated (see Figure 1), but is highlighted by the distribution of ants. The substrate tip is on the left. Change of colour intensity does not represent the exact number of ants, but serves to give an impression of general dynamics of the construction process. Figure 3 is included in the video as an indication of the colour scale. The initial exploratory phase is followed by groups forming at the tip and the bottom right of the substrate and successive rolling of the substrate tip. Original images were taken every 5 seconds and are displayed at 2 frames per second. Colour maps created with ImageJ.
(AVI)
